# Discovery of potent measles virus fusion inhibitor peptides *via* structure-guided derivatization†

**DOI:** 10.1039/d4md01006j

**Published:** 2025-01-24

**Authors:** Ziwei Gao, Jiei Sasaki, Tateki Suzuki, Tomoaki Suzuki, Yuki Miwa, Shinsuke Sando, Takao Hashiguchi, Jumpei Morimoto

**Affiliations:** a Depratement of Chemistry and Biotechnology, Graduate School of Engineering, The University of Tokyo Tokyo 113-8656 Japan ssando@chembio.t.u-tokyo.ac.jp jmorimoto@chembio.t.u-tokyo.ac.jp; b Laboratory of Medical Virology, Institute for Life and Medical Sciences, Kyoto University Kyoto 606-8507 Japan hashiguchi.takao.1a@kyoto-u.ac.jp; c Depratement of Bioengineering, Graduate School of Engineering, The University of Tokyo Tokyo 113-8656 Japan ssando@chembio.t.u-tokyo.ac.jp

## Abstract

Fusion inhibitor peptide (FIP), a short peptide known as a measles virus (MeV) infection inhibitor, inhibits membrane fusion between the viral envelope of MeV and the host cell membrane. Therefore, FIP is potentially useful as a drug candidate for treating MeV infection, but improvement of inhibitory activity is desirable. In this study, we conducted a structure–activity relationship study of FIP and, based on the result and the previously reported crystal structure of the complex, we designed FIP derivatives. From a series of derivatives, we discovered an FIP derivative with a strong inhibitory activity (IC_50_ = 210 nM) derived from the enhanced binding affinity (*K*_D_ = 6.6 nM) to the MeV fusion protein.

## Abstract 2

Structure-guided design led to the discovery of a small and potent peptide inhibitor of measles virus fusion protein.

## Graphical abstract

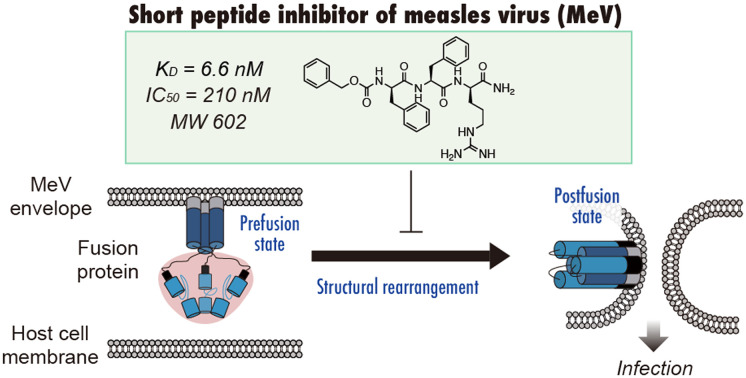

## Introduction

**Fig. 1:**
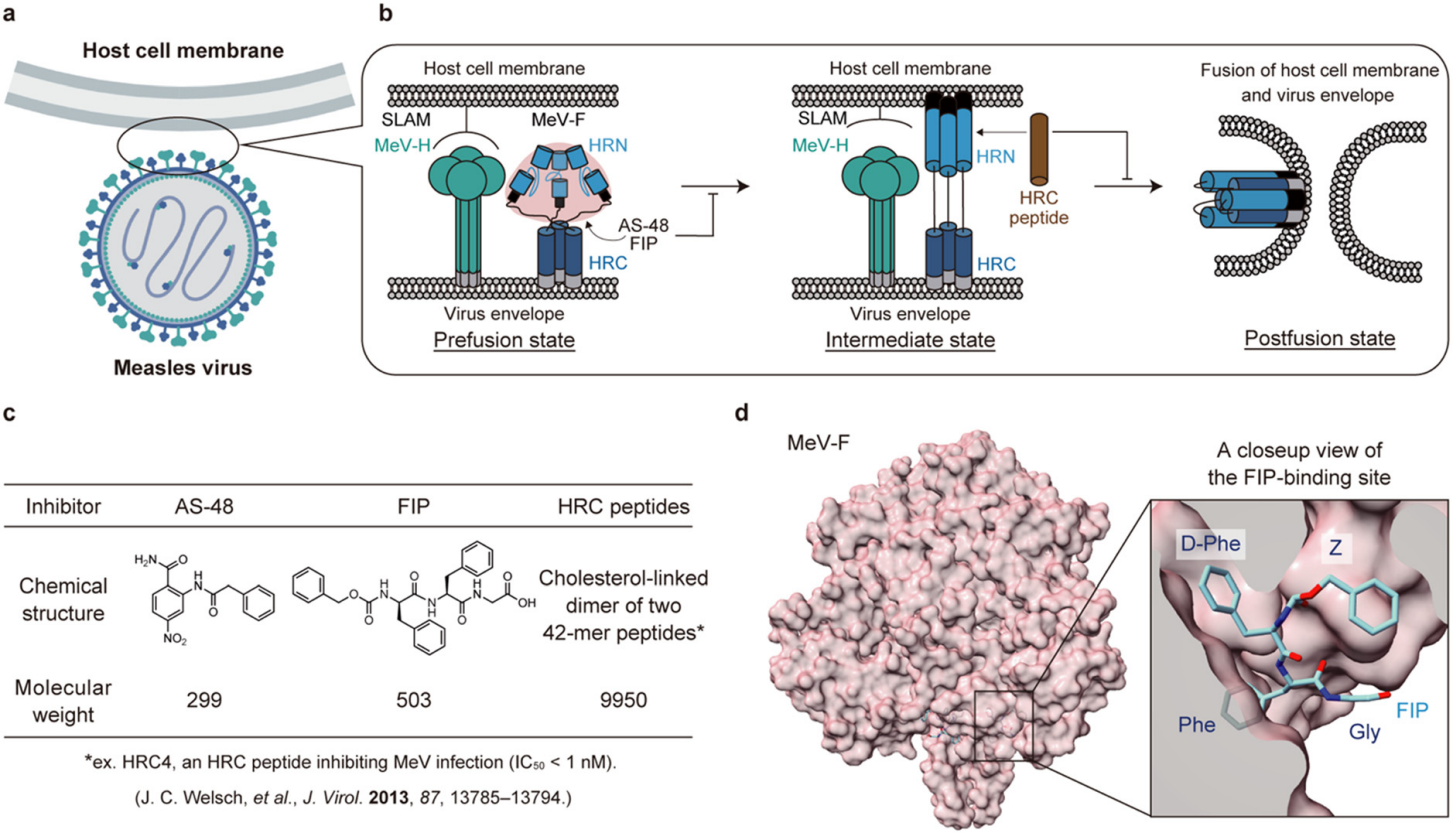
A schematic illustration of membrane fusion of measles virus (MeV) with the host cells and inhibition of the fusion. a) An illustration of MeV and a host cell. b) A schematic illustration of membrane fusion process of MeV with the host cell. MeV-H is colored green. The domains of MeV-F are colored as follows: HRN (blue), HRC (navy blue), fusion peptide (black), transmembrane (gray), and other parts of prefusion state MeV-F (rose pink). FIP and AS-48 bind and stabilize the prefusion state of MeV-F while the HRC peptide (brown), a mimic of the HRC domain, binds to the HRN domain of MeV-F and inhibits the conformational change to the postfusion state. c) Structures and molecular weights of MeV entry inhibitors binding to MeV-F. d) The crystal structure of the prefusion state of MeV-F bound with FIP (PDB ID: 5YZD).

Measles virus (MeV) is a highly contagious single-stranded negative-sense RNA virus (Fig. 1a). Despite the widespread use of vaccines, there were still an estimated 136 200 deaths due to MeV infection in 2022.^1^ In addition, the SARS-CoV-2 pandemic has delayed the measles vaccination program in developing countries, raising concerns about a recent outbreak.^2,3^ MeV infection not only leads to rare but fatal cases of measles inclusion body encephalitis (MIBE) and subacute sclerosing panencephalitis (SSPE), but also erases 11–73% of the antibody repertoire, inducing immune amnesia.^4^ However, there is still no approved drug.

Entry inhibitors are attractive as drug candidates of MeV with low side effects because they target membrane fusion pathways unique to viruses. Membrane fusion is triggered by two proteins on the MeV envelope: hemagglutinin (MeV-H) and fusion (MeV-F) proteins (Fig. 1a and b). First, MeV-H binds to receptors, such as signaling lymphocytic activation molecule (SLAM)^5^ and nectin-4^6,7^ on the host cell membrane, inducing a series of structural rearrangements of MeV-F (Fig. 1b, from left to middle). As a result, fusion peptide (FP) in MeV-F is exposed and inserted into the host cell membrane (Fig. 1b, middle). Then, the C-terminal helical heptad repeats (HRC) and the N-terminal helical heptad repeats (HRN) of MeV-F form a six-helix bundle structure, and MeV-F becomes a postfusion state (Fig. 1b, right). The conformational change of MeV-F results in the fusion of the MeV envelope with the host cell membrane, allowing MeV to enter the cell.^8^ MeV then replicates itself in the cell, budding and infecting other cells (virus-cell fusion).^9^ When infected, expression of MeV-H and MeV-F on the host cell membrane causes cell-to-cell fusion between the infected cell and adjacent cells, producing syncytia (cell-to-cell fusion).^10^ Thus, targeting MeV-H or MeV-F represents two distinct strategies for developing MeV entry inhibitors. Because certain MeV strains can infect neurons without SLAM or nectin-4,^11,12^ inhibition of the conformational change of MeV-F is a more promising strategy to inhibit MeV infection in cells than inhibition of the interaction between MeV-H and the receptors.

Two types of MeV entry inhibitors targeting MeV-F have been reported.^13–15^ The first type of inhibitor binds to the intermediate state of MeV-F and prevents the MeV-F conformational change to the postfusion state (Fig. 1b, middle). The representative inhibitors of this type are HRC peptides, which are based on and modified from the HRC domain of MeV-F (Fig. 1c). HRC peptides competitively bind to the HRN domain with the viral HRC domain, thereby preventing the MeV-F protein from transitioning to its postfusion state.^13,16^ Although HRC peptides exhibit high inhibitory activity,^13,17^ their large molecular weight (∼10 kDa) significantly compromises their stability *in vivo*, resulting in a short half-life (*t*_1/2_ < 1 h),^18^ and renders them unsuitable for oral administration. Even though modifying HRC peptides to self-assemble into nanoparticles can enhance their stability *in vivo*,^18^ this modification still does not make them viable for oral delivery.

The other type of MeV entry inhibitors are compounds binding to the prefusion state of MeV-F (Fig. 1b, left). A small molecule inhibitor, AS-48,^14^ and a short peptide inhibitor, fusion inhibitor peptide (FIP),^15^ are this type of inhibitors (Fig. 1c). The two compounds were recently shown to bind to a hydrophobic pocket between the head and stalk of MeV-F in the prefusion state (Fig. 1d). The inhibitor presumably stabilizes the prefusion state, thereby inhibiting the virus envelope fusion with the host cell membrane.^19^ Because both AS-48 and FIP are small in molecular size (Fig. 1c), they can potentially be inexpensive and orally available drugs. However, their inhibitory activities are low and need improvement.

Here, we report the design of FIP derivatives based on the crystal structure of MeV-F bound with FIP for producing potent entry inhibitors. We chose FIP as the lead compound for optimization because the peptidic inhibitor is easier to derivatize than the small molecule inhibitor, AS-48. We synthesized the designed compounds and evaluated their inhibitory activities.

## Results and discussion

### Structure–activity relationship of FIP

FIP is a short peptide with the sequence of Z-d-Phe-Phe-Gly (Z denotes carbobenzoxy group). The molecule was initially reported to have a half inhibitory concentration (IC_50_) of 0.2 μM in a plaque assay for MeV.^15^ However, in later reports, FIP has been used at 10 μM or higher concentration in virus infection assays or fusion assays using cultured mammalian cells, suggesting that IC_50_ of FIP is in μM order.^19,20^ Therefore, improvement of the inhibitory activity is needed to realize a drug, and we decided to design and synthesize FIP derivatives.

Upon designing FIP derivatives, we relied on the recently reported crystal structure of the prefusion state of MeV-F bound with FIP (Fig. 1d).^19^ In the crystal structure, most of the FIP structure is buried in the hydrophobic pocket of MeV-F, and all the aromatic rings in the FIP structure contact MeV-F. On the other hand, the C-terminal structure of FIP is exposed to solvent in the crystal structure. Based on the structural information, we hypothesized that the C-terminal region of FIP could be modified without disrupting its original interactions, potentially enabling the design of more potent inhibitors by introducing new interactions.

**Fig. 2:**
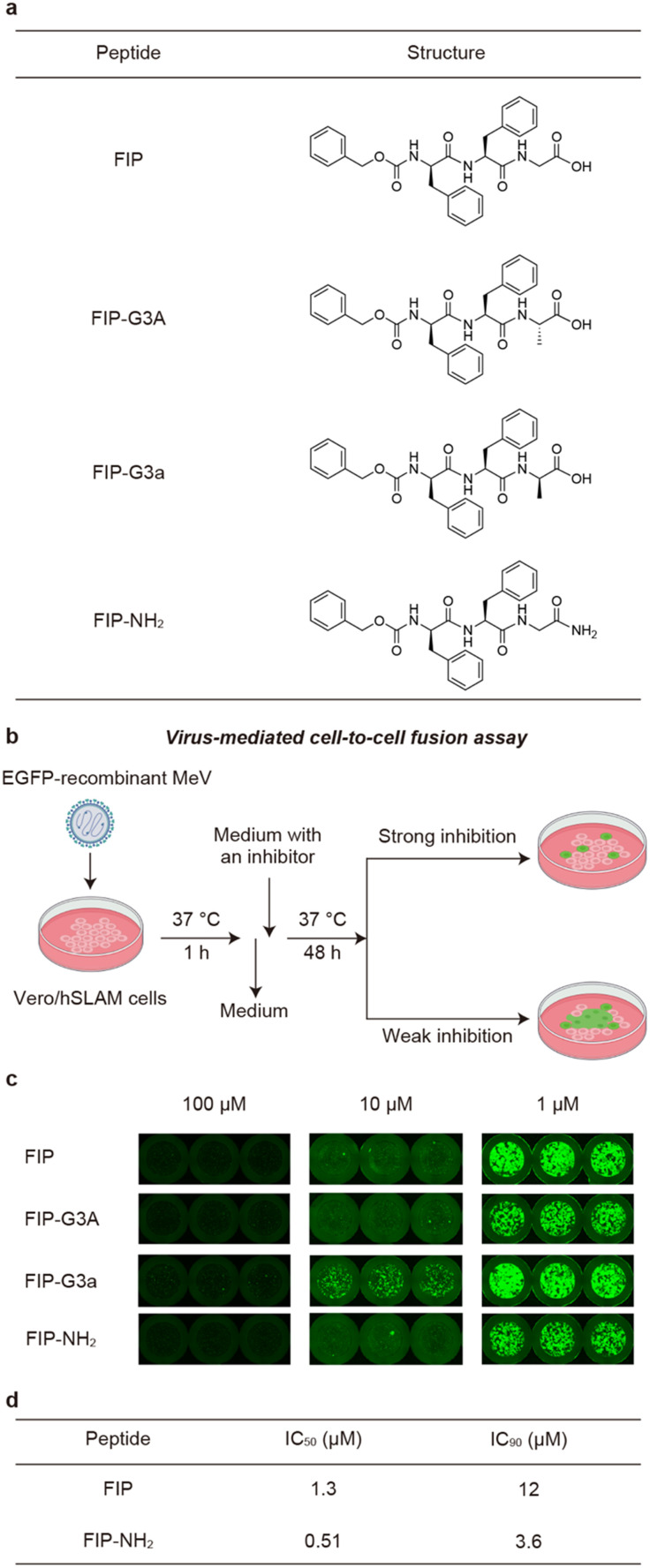
Investigation of the interactions between the C-terminus of FIP and MeV-F. a) Chemical structure of FIP, FIP-G3A, FIP-G3a and FIP-NH_2_. b) The scheme of virus mediated cell-to-cell fusion assay. c) Evaluation of the inhibitory activities of FIP, FIP-G3A, FIP-G3a, and FIP-NH_2_ against MeV-induced cell fusions. The degree of cell fusions was qualitatively assessed by the size and area of the syncytia emitting the fluorescence of EGFP that is encoded in the MeV. Cells were treated with 100, 10, or 1 μM of each compound. All the experiments were conducted in triplicate. d) Inhibitory activities of FIP and FIP-NH_2_ determined by viral entry inhibition assay.

To evaluate how modifying the C-terminus of FIP affects the interactions of FIP and MeV-F, we synthesized three FIP derivatives, named FIP-G3A, FIP-G3a, and FIP-NH_2_ (Fig. 2a). The inhibitory activities of the derivatives were evaluated using a virus-mediated cell-to-cell fusion assay (Fig. 2b). In this assay, Vero cells stably expressing hSLAM (Vero/hSLAM cells)^21^ were infected with EGFP-recombinant MeV (MeV-EGFP) for 1 h at 37 °C, then the medium containing MeV-EGFP was replaced with a medium containing each FIP derivative and incubated at 37 °C for 48 h. The inhibitory activity was assessed based on the size and area of the syncytia observed by fluorescence microscopy, albeit qualitatively. As a result, FIP strongly inhibited syncytia formation at 100 μM and 10 μM but inhibited fusion only weakly at 1 μM (Fig. 2c, FIP). This is consistent with the previous reports.^19,20^ Substitution of Gly in FIP with l-Ala or d-Ala did not largely change the inhibitory activity (Fig. 2c, FIP-G3A and FIP-G3a). This suggests that a functional group can be introduced as a side chain of the C-terminal Gly without strongly affecting the existing interactions. C-terminal amidation also did not decrease the inhibitory activity, which is consistent with the fact that the C-terminal carboxy group is not involved in the interaction with MeV-F in the previously reported crystal structure (Fig. 2c, FIP-NH_2_).^19^ FIP-NH_2_ showed a little higher inhibitory activity than FIP at 1 μM (Fig. 2c). To more quantitatively compare the inhibitory activity of FIP and FIP-NH_2_ against MeV infection in cells, a viral entry inhibition assay was conducted using a serially diluted compound, and the IC_50_ and IC_90_ values of the compounds were determined. In this assay, Vero/hSLAM cells were infected with inhibitor-treated MeV-EGFP at 37 °C for 1 h. Subsequently, the supernatant was replaced with a medium containing 100 μM of FIP, and the cells were further incubated at 37 °C for 48 h. Finally, the number of virus-infected cells was quantified using fluorescence microscopy. As a result, FIP-NH_2_ showed a slightly higher viral entry inhibitory activity (IC_50_ = 0.51 μM, IC_90_ = 3.6 μM) than FIP (IC_50_ = 1.3 μM, IC_90_ = 12 μM) (Fig. 2d and S1†). Based on these results, we assumed that derivatization of the C-terminal Gly moiety of FIP could create a new interaction between FIP and MeV-F without compromising the interaction formed between FIP and MeV-F, thereby improving the inhibitory activity. Since FIP-NH_2_ showed a little higher inhibitory activity than FIP, we decided to use FIP-NH_2_ as the lead structure for further derivatization and introduce various functional groups at the α-carbon of the C-terminal Gly residue of FIP-NH_2_.

### Derivatization of FIP at the C-terminus

**Fig. 3:**
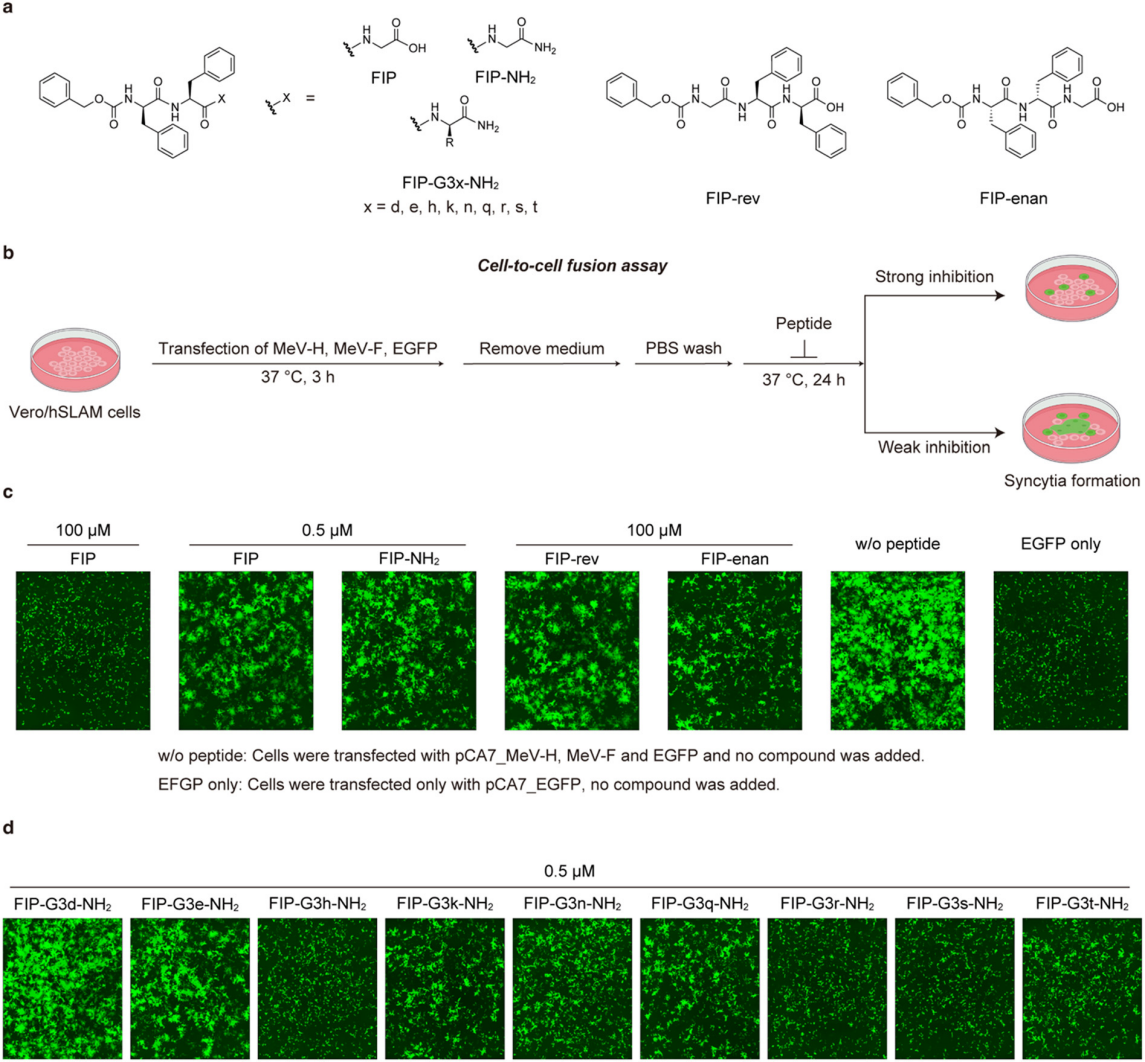
Chemical structures and cell-to-cell fusion assay of FIP and its derivatives using Vero/hSLAM cells expressing MeV-F and MeV-H. a) Chemical structures of FIP and its derivatives. b) A schematic illustration of cell-to-cell fusion assay. The degree of cell fusion inhibitory activity was qualitatively assessed based on the size and area of the syncytia emitting the EGFP fluorescence in Vero/hSLAM cells. c) The cells treated with FIP, FIP-NH_2_, FIP-rev, and FIP-enan are shown. Control cells that are not treated with a peptide (w/o peptide) and that are transfected with only EGFP plasmid (EGFP only) are also shown. d) The results of the cells treated with 0.5 μM FIP-NH_2_ derivatives. All the experiments were conducted in triplicate. The other two results are shown in Fig. S2.†

To obtain new interactions, we designed and synthesized FIP-NH_2_ derivatives in which Gly residue is replaced with a d-amino acid residue shown in Fig. 3a (FIP-G3*x*-NH_2_, *x* = d, e, h, k, n, q, r, s, t). To avoid proteolytic degradation of the derivatized peptides, d-amino acid residues were adopted. Because FIP consists of hydrophobic structures, polar residues were recruited for replacing the Gly residue to avoid poor aqueous solubility of the resulting derivatives. The inhibitory activities of the synthesized derivatives were evaluated by cell-to-cell fusion assay. In this assay, each FIP derivative was added to the medium of Vero/hSLAM cells transiently expressing MeV-F, MeV-H, and EGFP, and the cells were incubated at 37 °C for 24 h. The cells were observed under a fluorescence microscope, and inhibition of the syncytia formation was qualitatively assessed based on the size and area of emitting fluorescence (Fig. 3b). We also synthesized and evaluated the reverse sequence of FIP, FIP-rev (Z-Gly-Phe-d-Phe-OH), and the enantiomer of FIP, FIP-enan (Z-Phe-d-Phe-Gly-OH), as negative controls.

As a result, first, FIP strongly inhibited syncytia formation at 100 μM and weakly inhibited syncytia formation at 0.5 μM (Fig. 3c and S2†). FIP-NH_2_ inhibited syncytia formation to a similar degree or slightly more strongly compared to FIP at 0.5 μM. These results are consistent with the result of the virus-mediated cell-to-cell fusion assay in Fig. 2c. In contrast, the control compounds, FIP-rev and FIP-enan, showed lower inhibitory activity than FIP at 100 μM. This result supports the specificity of the interaction between FIP and MeV-F.

Among the 9 FIP-NH_2_ derivatives, FIP-G3d-NH_2_ and FIP-G3e-NH_2_ exhibited weak or no inhibitory activity at 0.5 μM. On the other hand, the other 7 derivatives, FIP-G3h-NH_2_, FIP-G3k-NH_2_, FIP-G3n-NH_2_, FIP-G3q-NH_2_, FIP-G3r-NH_2_, FIP-G3s-NH_2_, and FIP-G3t-NH_2_ exhibited higher inhibitory activity than FIP-NH_2_. Among the 7 derivatives, FIP-G3h-NH_2_, FIP-G3r-NH_2_, and FIP-G3s-NH_2_ exhibited the highest inhibitory activity. These FIP derivatives completely inhibited the syncytia formation at 0.5 μM (Fig. 3d and S2†). We chose FIP-G3r-NH_2_ among the three derivatives with the highest inhibitory activities for further study.

To quantitatively assess the inhibitory activity of FIP-G3r-NH_2_ against MeV infection in cells, we performed a viral entry inhibition assay. We also performed the same assay for FIP as a comparison. As a result, the IC_50_ value of FIP-G3r-NH_2_ was 0.21 μM (IC_90_ = 1.8 μM) (Table 1 and Fig. S1†), which is about six-fold higher inhibitory activity than that of FIP (IC_50_ = 1.3 μM).

**Table 1:** Inhibitory activity and binding affinity of FIP and FIP-G3r-NH_2_

	FIP-NH_2_	FIP-G3r-NH_2_
Structure	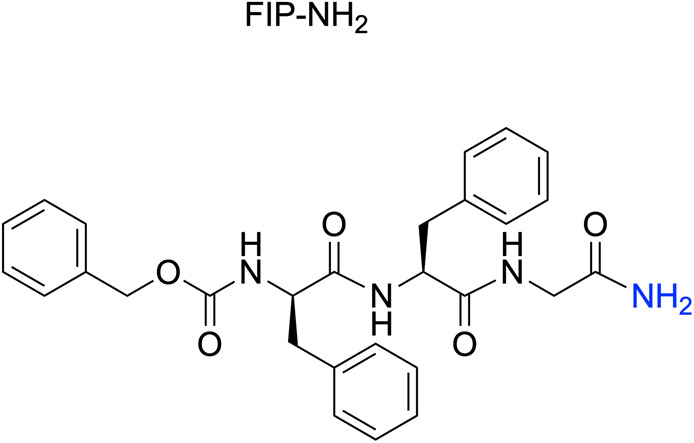	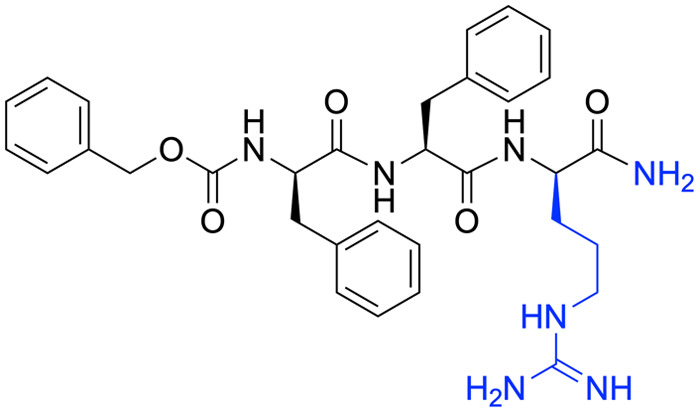
IC_50_ (μM)^a^	0.51	0.21
IC_90_ (μM)^a^	3.6	1.8
*K*_D_ (nM)^b^	81	6.6

a The value was obtained from the viral entry inhibition assay. The experiment was conducted in triplicate.

b Peptides were immobilized on sensor chip and MeV-F was used as the analyte.

### Investigation of plausible reasons for the improved inhibitory activity

The enhanced inhibitory activity of FIP-G3r-NH_2_ can be attributed to its ability to form new interactions with MeV-F. Therefore, we evaluated the binding affinity of FIP-G3r-NH_2_ to MeV-F and compared it with that of FIP-NH_2_. Peptide derivatives containing an ethylene glycol spacer and a Lys residue at the C-terminus (FIP-NH-EG-K and FIP-G3r-NH-EG-K) were synthesized for immbilization on a sensor chip used in surface plasmon resonance (SPR) measurements (the peptide structures are provided in Fig. S3†). The derivatized peptides were immobilized on the sensor chip, and their binding affinities to MeV-F were evaluated using SPR (Table 1 and Fig. S3†). To isolate MeV-F for the binding experiment, its ectodomain (composed of the head and stalk) trimer was stabilized in the prefusion state by introducing cysteine substitutions into the stalk region, as demonstrated in the previous study.^19^ The dissociation constant (*K*_D_) for MeV-F was determined to be 81 nM for FIP-NH-EG-K, corresponding to FIP-NH_2_, and 6.6 nM for FIP-G3r-NH-EG-K, corresponding to FIP-G3r-NH_2_, representing a 12-fold improvement over FIP-NH_2_ (Table 1). The improvement in affinity is consistent with the enhancement in the inhibitory activity of FIP-G3r-NH_2_ over FIP-NH_2_. This result suggests that FIP-G3r-NH_2_ binds more strongly with MeV-F and stabilizes its prefusion state more effectively than FIP and FIP-NH_2_, leading to higher inhibitory activity against MeV-F-mediated viral entry into mammalian cells (Table 1).

**Fig. 4:**
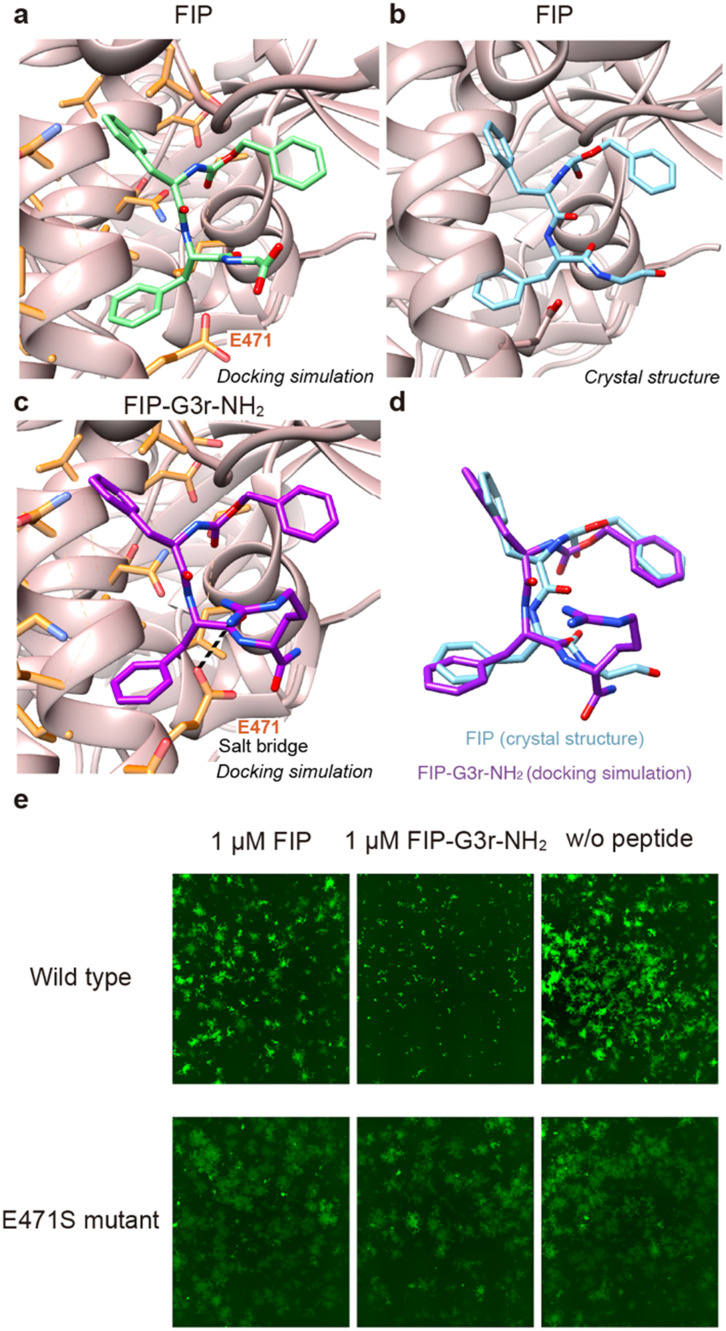
Investigation of the plausible interaction between the guanidium group of FIP-G3r-NH_2_ and MeV-F. a) A docking pose of FIP with MeV-F. FIP and MeV-F are colored in green and rose pink. b) The previously reported crystal structure of FIP bound to MeV-F (PDB ID: 5YZD). FIP is colored in cyan. c) A docking pose of FIP-G3r-NH_2_ with MeV-F. FIP-G3r-NH_2_ is colored in purple. A salt bridge between FIP-G3r-NH_2_ and the E471 residue of MeV-F is indicated by a dashed line. In Fig. 4a and c, MeV-F residues that shifted during the docking simulations are shown in orange. d) Comparison of the structure between FIP and FIP-G3r-NH_2_. e) Evaluation of the inhibitory activity of FIP and FIP-G3r-NH_2_ against MeV-F wild type (WT) and the E471S mutant by cell-to-cell fusion assay. All the experiments were conducted in triplicate. The other two results at 1 μM and the results at other concentration are shown in Fig. S4.†

To identify the plausible new interactions formed between FIP-G3r-NH_2_ and MeV-F, we conducted docking simulations using Autodock Vina.^22,23^ First, we conducted a docking simulation of FIP and MeV-F to assess the reliability of the docking simulations (Fig. 4a). As a result, we observed docking poses in which FIP binds to MeV-F in a similar orientation to that in the previously reported crystal structure among the top 20 poses (Fig. 4b), suggesting the validity of the docking simulations. We then conducted a docking simulation of FIP-G3r-NH_2_ and MeV-F (Fig. 4c). We found a docking pose in which the binding conformation of the main chain and the aromatic ring of FIP-G3r-NH_2_ is similar to that of FIP (Fig. 4d). We used PLIP (protein–ligand interaction profiler)^24^ to analyze the interaction between the docked structure of FIP-G3r-NH_2_ and MeV-F. The analysis suggests that the guanidinium group unique to FIP-G3r-NH_2_ forms a salt bridge with the E471 residue of MeV-F. Since the introduction of the guanidinium group increased the inhibitory activity of FIP more significantly than the modification of the C-terminal carboxylate to an amide, the new interaction formed by the guanidinium group is considered to play a major role in enhancing the activity. To experimentally evaluate whether the salt bridge between the guanidinium group and the E471 residue contributes to enhancing the inhibitory activity, we evaluated the inhibitory activity of FIP and FIP-G3r-NH_2_ against the E471S mutant of MeV-F. As a result, while FIP-G3r-NH_2_ exhibited a higher membrane fusion inhibitory activity than FIP for the wild-type (WT), the inhibitory activity of FIP-G3r-NH_2_ against the E471S mutant of MeV-F dropped to the same level as FIP (Fig. 4e and S4†). These results support the validity of the docking simulation result that the newly acquired salt bridge between the guanidium group of FIP-G3r-NH_2_ and E471 of MeV-F plays a role in enhancing the inhibitory activity. It should be noted that the MeV-F E471S mutant protein was synthetically produced for this experiment, and, to our knowledge, this mutant has not been reported as a natural variant. This implies that, for the time being, the mutational escape of MeV-F from FIP-G3r-NH_2_ needs not be a concern.

The higher inhibitory activity of FIP-G3r-NH_2_ than FIP could also be due to improved proteolytic stability, aqueous solubility, and/or reduced adsorption to serum proteins under cell culture conditions. To assess the degree of the contribution from these factors, first, we evaluated the stability of each 1 μM inhibitor in serum. Both FIP and FIP-G3r-NH_2_ showed high proteolytic stability (Table 2 and Fig. S5†). Therefore, the proteolytic stability is not involved in the observed difference in the inhibitory activities between FIP and FIP-G3r-NH_2_. Next, the solubility of FIP and FIP-G3r-NH_2_ in 1% DMSO/PBS was examined and found to be 98 ± 1 and 83 ± 4 μM, respectively. This result shows that both inhibitors are almost completely dissolved in the experimental conditions for the cell-to-cell fusion assay (0.5 μM) and viral entry inhibition assay (Table 2). Therefore, the enhanced inhibitory activity of FIP-G3r-NH_2_ does not relate to the improvement in solubility. On the other hand, when the adsorption of 1 μM inhibitor on serum proteins in 10% FBS/PBS was evaluated, FIP-G3r-NH_2_ was found to be less adsorbed on serum than FIP (Table 2 and Fig. S6†). The result indicates that the effective concentration of FIP-G3r-NH_2_ is higher than that of FIP in the serum-containing environment used for fusion assays. This can be one of the reasons for the higher inhibitory activity of FIP-G3r-NH_2_. However, this is probably not the major reason for the improvement of the inhibitory activity, considering that the difference in the effective concentration between the two compounds is less than two-fold. These results suggest that FIP-G3r-NH_2_ has a higher inhibitory activity than FIP due to the new interaction with MeV-F and increased effective concentration.

**Table 2:** Physicochemical parameters of FIP and FIP-G3r-NH_2_

	FIP	FIP-G3r-NH_2_
*t*_1/2_ in 10% FBS/PBS (h)^a^	>24	>24
Solubility (μM)^b^	98 ± 1	83 ± 4
Serum adsorption (%)^c^	34 ± 2	3 ± 4

a 1 μM inhibitor in 1% DMSO/10% FBS/PBS, in triplicate.

b 100 μM inhibitor in 1% DMSO/PBS, in triplicate.

c 1 μM inhibitor in 1% DMSO/10% FBS/PBS. The assays with FIP and FIP-G3r-NH_2_ were conducted in quintuplicate and quadruplicate, respectively. [C11K]tigerinin-1*R*, which is susceptible to serum degradation, was used as a control to confirm the proteolytic activity of serum.

## Conclusions

In this study, based on the crystal structure of MeV-F bound with FIP, we designed, synthesized, and evaluated FIP derivatives to produce potent inhibitors against MeV infection. Among the derivatives with enhanced inhibitory activities, FIP-G3r-NH_2_ was investigated in detail and was shown to exhibit a high inhibitory activity. Computational and experimental investigations suggested that the inhibitory activity is enhanced by new interaction with MeV-F and increased effective concentration in solution.

Targeting the hydrophobic pocket of the prefusion state of the fusion protein is a promising strategy for producing membrane fusion inhibitors with small molecular sizes. In this study, a small modification to the lead compound, FIP, led to the discovery of a potent inhibitor targeting the hydrophobic pocket of MeV-F. The resultant modified inhibitor, FIP-G3r-NH_2_, exhibits strong binding affinity (*K*_D_ = 6.6 nM) and potent inhibitory activity (IC_50_ = 0.21 μM) while maintaining a small molecular weight (MW 602). Previously, two small molecules with a similar mechanism to FIP were reported as inhibitors for other viruses. They effectively stabilize the prefusion states of the respiratory syncytial virus's fusion protein^25^ and the influenza A virus's hemagglutinin.^26^ The previous reports and our study highlight the potential of targeting this specific binding pocket as a strategy to develop highly potent, low-molecular-weight inhibitors for combating virus infections. Our study not only demonstrates the potential of FIP-G3r-NH_2_ as a drug candidate for MeV infection but also emphasizes the feasibility of realizing potent inhibitors with small molecular size by targeting the prevalent hydrophobic pocket of a virus fusion protein. Since these small peptide inhibitors work with a different mechanism from well-known HRC peptides,^17^ combinatorial usage of the two types of inhibitors would be useful for more potently inhibiting virus infection (Fig. 1b).

## Data availability

The data supporting this article have been included as part of the ESI.† Materials and methods, along with additional figures and tables including the analytical liquid chromatography data of the synthesized compounds are available in ESI.†

## Author contributions

T. H. and J. M. conceptualized and designed the study. Z. G. and J. S. developed the methodology, performed data analysis, and carried out formal data analysis. Z. G., J. S., Tomoaki S., and Y. M. carried out the investigation and data collection. S. S., T. H., J. M., and Tateki S. provided resources and materials required for the study. Z. G. and J. M. prepared the original draft and figures of the manuscript. All the authors contributed to the review and edit of the manuscript. J. M., T. H., S. S., and Tateki S. supervised and managed the research work.

## Conflicts of interest

There are no conflicts to declare.

## Supplementary Material

MD-016-D4MD01006J-s001

MD-016-D4MD01006J-s002

MD-016-D4MD01006J-s003

MD-016-D4MD01006J-s004
