# Supplementary material for: Discovery of potent measles virus fusion inhibitor peptides via structure-guided derivatization
Source: RSC Med Chem. 2025 Jan 24;16(4):1619–25. doi: 10.1039/d4md01006j (PMC11799930; doi:10.1039/d4md01006j)
Supplement: MD-016-D4MD01006J-s004 [file MD-016-D4MD01006J-s004.pdf]

Supplementary Information (SI) for RSC Medicinal Chemistry.
This journal is © The Royal Society of Chemistry 2025

Supplementary Information

Discovery of potent measles virus fusion inhibitor peptides via structure-guided derivatization

Ziwei Gao, Jiei Sasaki, Tateki Suzuki, Tomoaki Suzuki, Yuki Miwa, Shinsuke Sando,* Takao Hashiguchi,* and Jumpei Morimoto*

Table of Contents

Materials and Methods 3

Abbreviations 3

General remarks 3

General procedures for the synthesis of FIP derivatives 3

Cell culture 8

Viral entry inhibition assay 8

Virus-mediated cell-to-cell fusion assay. 9

Cell-to-cell fusion assay using wild-type MeV-F plasmid 9

Cell-to-cell fusion assay using MeV-F E471S mutant 9

Surface plasmon resonance measurement (SPR) 9

Docking simulations 10

Stability in serum 10

Serum adsorption 10

Supplementary Figures 12

Figure S1. 12

Figure S2. 13

Figure S3. 14

Figure S4. 15

Figure S5. 16

Figure S6. 17

Figure S7. 18

References 19

Materials and Methods

Abbreviations

CTC, chlorotrityl chloride; MeCN, acetonitrile; DMF, *N*,*N*-dimethylformamide; DCM, dichloromethane; NMP, *N*-methyl-2-pyrrolidone； Z, benzyloxycarbonyl; Boc, *tert*-butoxycarbonyl; Trt, trityl; Pbf, 2,2,4,6,7-pentamethyldihydrobenzofuran-5-sulfonyl; *t*Bu, *tert*-butyl; HOAt, 3*H*-1,2,3-triazolo[4,5-*b*]pyridin-3-ol; DIPEA, *N*,*N*-diisopropylethylamine; COMU, ethyl 2-cyano-2-((dimethyliminio)(morpholino)methyloxyimino)acetate hexafluorophosphate; HATU, 1-[bis(dimethylamino)methylene]-1*H*-1,2,3-triazolo[4,5-*b*]pyridinium 3-oxide hexafluorophosphate; TFA, trifluoroacetic acid; TIPS, triisopropylsilane; FBS, fetal bovine serum; DMEM, Dulbecco's Modified Eagle Medium.

General remarks. All chemicals and solvents used in this study were purchased from commercial suppliers and used without further purification. Preparative HPLC was performed on a Prominence HPLC system (Shimadzu) with a 5C18-AR-II column (Nacalai tesque, 10 mm I.D.×150 mm, 34350-41). Analytical ultra-high performance liquid chromatography (UHPLC) was performed on a Nexera-i (Shimadzu) with a Shim-pack Velox C18 column (Shimadzu, 1.8 μm, 2.1 mm×50 mm, 22732007-02). Ultra-performance liquid chromatography (UPLC) and liquid chromatography-mass spectrometry (LC-MS) were performed on an ACQUITY UPLC H-Class/SQD2 (Waters) using ACQUITY UPLC BEH C18 column (Waters, 2.1 mm I.D. × 50 mm). FIP was purchased from PEPTIDE INSTITUTE. INC.

General procedures for the synthesis of FIP derivatives. Peptides were synthesized by standard solid-phase peptide synthesis procedures.

a) Synthesis on CTC resin.

   CTC resin was swelled in DCM for 10 min, and the solvent was removed. The resin was treated with DCM solution of Fmoc-Ala-OH or Fmoc-D-Ala-OH (4 eq., 0.2 M), with DIPEA（8 eq., 0.4 M) with continuous shaking for 2 h at RT. After the solution was removed, the resin was washed with DCM three times. Then, the resin was treated with 2 mL of 1/2/17 = DIPEA/methanol/DCM solution for 10 min at RT. After the solution was removed, the resin was washed with DCM and DMF three times each. To remove the Fmoc-protecting group, the resin was treated with 20% piperidine solution in DMF with continuous shaking for 3 min. After the solution was removed, the resin was washed with DMF once. Then, the resin was treated with 20% piperidine in DMF solution with continuous shaking for 12 min. After the solution was removed, the resin was washed with DMF three times. Then, the resin was treated with DMF solution of Fmoc-Phe-OH (4 eq., 0.2 M), COMU (4 eq., 0.2 M), and DIPEA (8 eq., 0.4 M) with continuous shaking for 40 min at RT. After the deprotection of Fmoc group, the resin was treated with DMF solution of Z-D-Phe (4 eq., 0.2 M), COMU (4 eq., 0.2 M), and DIPEA (8 eq., 0.4 M) with continuous shaking for 40 min. After the synthesis on solid-phase, peptides were cleaved by treating the resin with 1 mL of 95/2.5/2.5 = TFA/water/TIPS with continuous shaking for 2–3 h. The solution was transferred to a glass recovery flask. The resin was washed with 1 mL methanol twice, and the methanol was collected in the same flask. The solvent in

the flask was removed under reduced pressure. The crude product was dissolved in water and acetonitrile and purified using a reversed-phase column on HPLC, using water and acetonitrile containing 0.1% TFA as mobile phases. The purified product was analyzed by LC-MS and UPLC.

b) Synthesis on Rink Amide MBHA resin.
Peptides were synthesized by standard Fmoc solid phase peptide synthetic method on a fully automated parallel peptide synthesizer Syro I (Biotage) using Rink Amide-MBHA resin (#A00172, Watanabe Chemical Industries) and Fmoc-amino acids. Coupling reaction was performed with NMP solution of Fmoc-amino acid (4 eq., 0.2 M), HOAt (4 eq., 0.2 M), HATU (4 eq., 0.2 M), and DIPEA (8 eq., 0.4 M). After the final amino acid coupling reaction, the Fmoc-protecting group was removed by treating the resin with a 20% piperidine solution in DMF under continuous shaking for 3 minutes. After the solution was drained, the resin was washed once with DMF. The resin was then treated again with 20% piperidine in DMF solution under continuous shaking for 12 minutes. Following the removal of the solution, the resin was washed three times with DMF. The acetylation of [C11K]tigerinin-1R was performed manually. DMF solution of acetic anhydride (10 eq., 0.5 M) and pyridine (20 eq., 1 M) was added to the resin with continuous shaking for 3 h at RT. After removing the solution, the resin was washed with DMF three times. Then, peptides were cleaved by treating the resin with 1 mL of 95/2.5/2.5 = TFA/water/TIPS with continuous shaking for 2–3 h. The solution was transferred to a 15 mL centrifuge tube, then the resin was washed with 1 mL methanol twice, and the methanol was collected in the same tube; then, the solvent in the tube was removed under a reduced pressure. The crude product was dissolved in water and acetonitrile and then purified using a reversed-phase column on HPLC, using water and acetonitrile with 0.1% TFA as mobile phases. The purified product was analyzed by LC-MS and UPLC.

Synthesis of FIP-G3A.

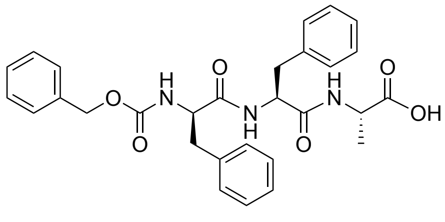

125 mg of CTC resin (1.69 mmol/g, 211.3 μmol) was used for synthesis. Yield was 2.2 mg (2%). ESI-MS *m/z*: $[M+H]^+$ Calcd for $C_{29}H_{32}N_3O_6^+$ 518.2; Found 518.5.

Synthesis of FIP-G3a.

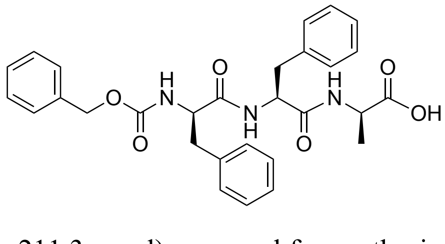

125 mg of CTC resin (1.69 mmol/g, 211.3 μmol) was used for synthesis. Yield was 1.4 mg (0.7%). ESI-MS *m/z*: $[M+H]^+$ Calcd for $C_{29}H_{32}N_3O_6^+$ 518.2; Found 518.6.

Synthesis of FIP-NH$_2$.

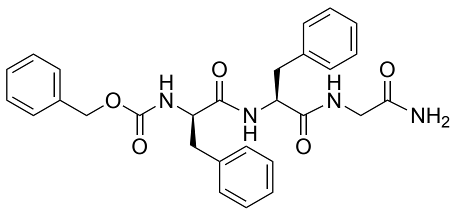

192 mg of Rink Amide MBHA resin (0.52 mmol/g, 100 μmol) was used for synthesis. Yield was 28.6 mg (57%). ESI-MS *m/z*: $[M+Na]^+$ Calcd for $C_{28}H_{30}N_4NaO_5^+$ 525.2; Found 525.6.

Synthesis of FIP-G3d-NH$_2$.

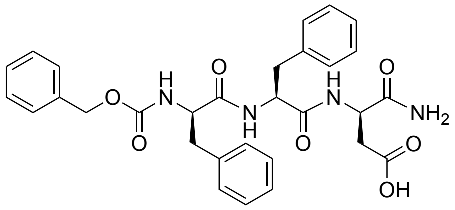

192 mg of Rink Amide MBHA resin (0.52 mmol/g, 100 μmol) was used for synthesis. Yield was 11.9 mg (21%). ESI-MS *m/z*: $[M-H]^+$ Calcd for $C_{30}H_{31}N_4O_7^+$ 559.2; Found 559.4.

Synthesis of FIP-G3e-NH$_2$.

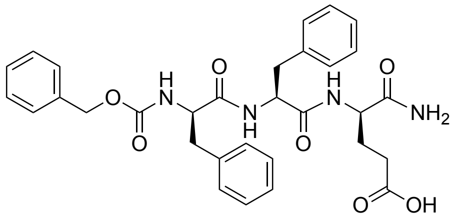

192 mg of Rink Amide MBHA resin (0.52 mmol/g, 100 μmol) was used for synthesis. Yield was 10.7 mg (17%). ESI-MS *m/z*: $[M-H]^+$ Calcd for $C_{31}H_{33}N_4O_7^+$ 573.2; Found 573.5.

Synthesis of FIP-G3h-NH$_2$.

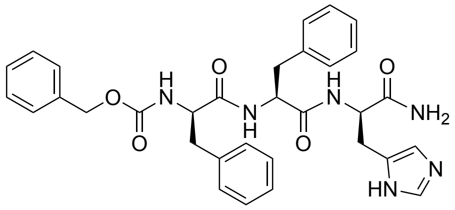

192 mg of Rink Amide MBHA resin (0.52 mmol/g, 100 μmol) was used for synthesis. Yield was 4.4 mg (8%). ESI-MS *m/z*: $[M+H]^+$ Calcd for $C_{32}H_{35}N_6O_5^+$ 583.3 Found 583.5.

Synthesis of FIP-G3k-NH$_2$.

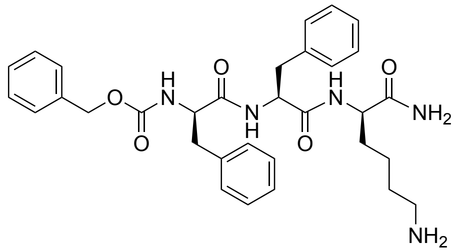

192 mg of Rink Amide MBHA resin (0.52 mmol/g, 100 μmol) was used for synthesis. Yield was 33.3 mg (58%). ESI-MS *m/z*: $[M+H]^+$ Calcd for $C_{32}H_{40}N_5O_5^+$ 574.3; Found 574.6.

Synthesis of FIP-G3n-NH₂.

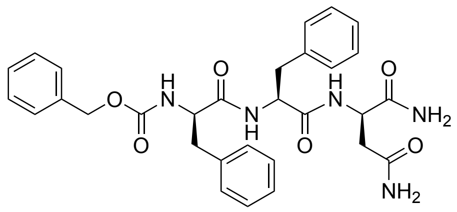

192 mg of Rink Amide MBHA resin (0.52 mmol/g, 100 μmol) was used for synthesis. Yield was 17.8 mg (32%). ESI-MS *m/z*: $[M+Na]^+$ Calcd for $C_{30}H_{34}N_5O_6^+$ 560.2; Found 560.5.

Synthesis of FIP-G3q-NH₂.

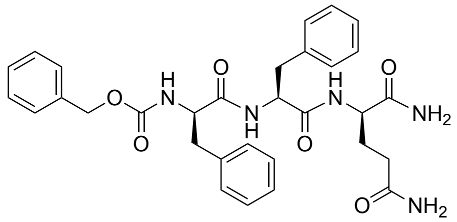

141 mg of Rink Amide MBHA resin (0.71 mmol/g, 100 μmol) was used for synthesis. Yield was 21.6 mg (38%). ESI-MS *m/z*: $[M+H]^+$ Calcd for $C_{31}H_{36}N_5O_6^+$ 574.3; Found 574.5.

Synthesis of FIP-G3r-NH₂.

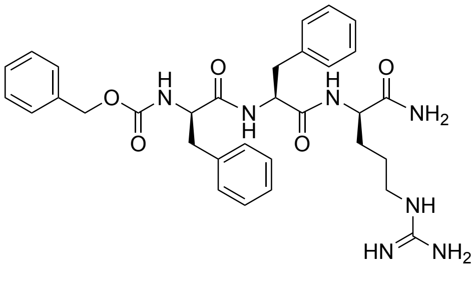

141 mg of Rink Amide MBHA resin (0.71 mmol/g, 100 μmol) was used for synthesis. Yield was 31.7mg (53%). ESI-MS *m/z*: $[M+H]^+$ Calcd for $C_{32}H_{40}N_7O_5^+$ 602.3; Found 602.6.

Synthesis of FIP-G3s-NH₂.

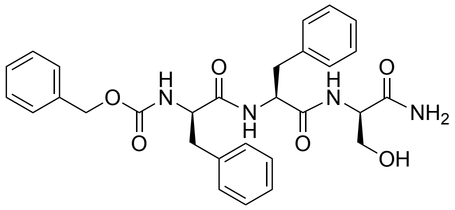

141 mg of Rink Amide MBHA resin (0.71 mmol/g, 100 μmol) was used for synthesis. Yield was 21.6 mg (41%). ESI-MS *m/z*: $[M+Na]^+$ Calcd for $C_{29}H_{32}N_4NaO_6^+$ 555.2; Found 555.5.

Synthesis of FIP-G3t-NH$_2$.

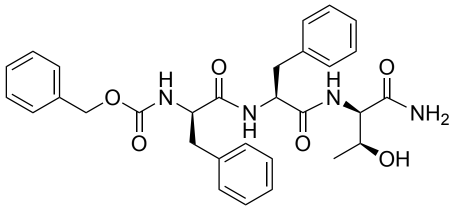

192 mg of Rink Amide MBHA resin (0.52 mmol/g, 100 μmol) was used for synthesis. Yield was 12.6 mg (23%). ESI-MS *m/z*: $[M+Na]^+$ Calcd for $C_{30}H_{34}N_4NaO_6^+$ 569.2; Found 569.5.

Synthesis of FIP-rev.

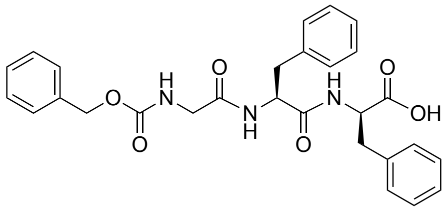

227.3 mg of CTC resin (1.32 mmol/g, 300 μmol) was used for synthesis. Yield was 74.1 mg (49%). ESI-MS *m/z*: $[M+H]^+$ Calcd for $C_{28}H_{30}N_3O_6$ 504.2; Found 504.5.

Synthesis of FIP-enan.

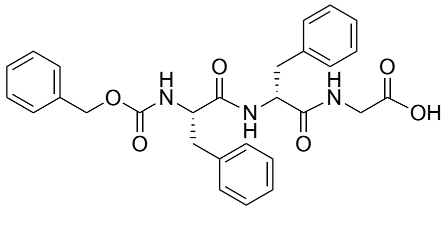

227.3 mg of CTC resin (1.32 mmol/g, 300 μmol) was used for synthesis. Yield was 63.3 mg (42%). ESI-MS *m/z*: $[M+Na]^+$ Calcd for $C_{28}H_{29}N_3NaO_6^+$ 526.2; Found 526.5.

Synthesis of [C11K]tigerinin-1R.

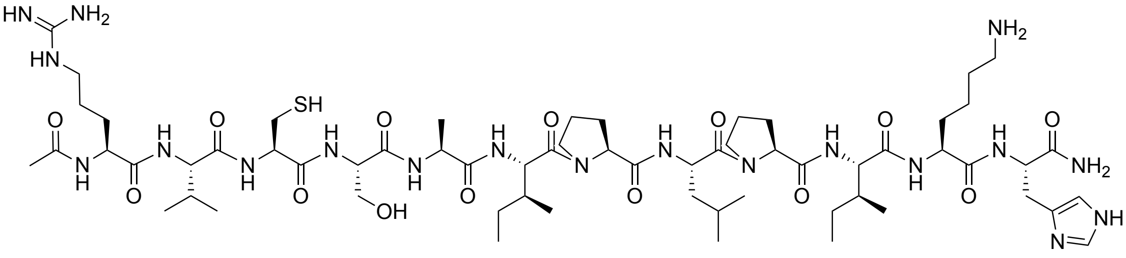

122 mg of Rink Amide MBHA resin (0.41 mmol/g, 50 μmol) was used for synthesis. Yield was 3.4 mg (5%). ESI-MS *m/z*: $[M+2H]^{2+}$ Calcd for $C_{62}H_{109}N_{19}O_{14}S^{2+}$ 687.9; Found 688.4.

Synthesis of FIP-NH-EG-K.

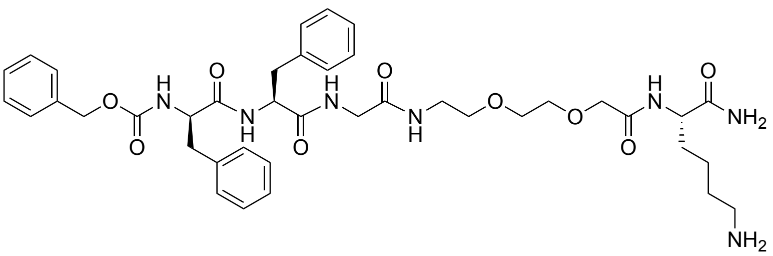

54 mg of Rink Amide MBHA resin (0.46 mmol/g, 25 μmol) was used for synthesis. Yield was 8.7 mg (45%). LC-MS *m/z*: $[M+H]^{+}$ Calcd for $C_{40}H_{54}N_{7}O_{9}$ 776.4; Found 777.1.

Synthesis of FIP-G3r-NH-EG-K.

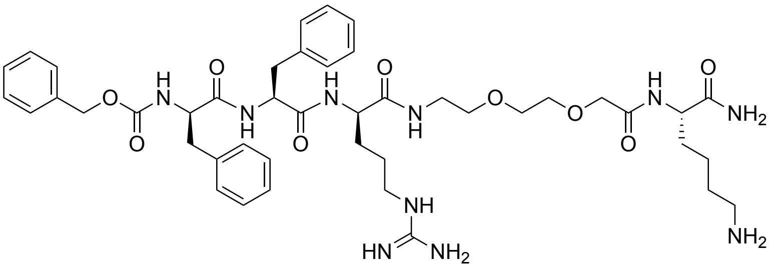

54 mg of Rink Amide MBHA resin (0.46 mmol/g, 25 μmol) was used for synthesis. Yield was 6.3 mg (29%). LC-MS *m/z*: $[M+H]^{+}$ Calcd for $C_{44}H_{63}N_{10}O_{9}$ 875.5; Found 875.7.

Cell culture. Vero cells expressing hSLAM (Vero/hSLAM)[1] were cultured in DMEM supplemented with 10% fetal bovine serum (FBS), 1% Penicillin-Streptomycin and 1% G418.

Plasmid. Full-length E471S MeV-F was amplified through PCR and site-directed mutagenesis. The template used was p(+)MV323, which houses the antigenomic full-length cDNAs of the IC-B strain MeV-F[2]. The fragment was then cloned into the pCA7 vector[3]. The expression plasmids for the EGFP (pCA7-EGFP), the ectodomain of the MeV F-protein (T20 to V496), and wild-type full-length MeV-H (pCA7-ICH) and MeV-F (pCA7-ICF) had been described in the previous reports[4–6].

Viral entry inhibition assay. Vero/hSLAM cells were cultured in 96 well plates (Thermo scientific cat#167008) overnight at 37 °C. EGFP-recombinant MeV IC323 strain (MeV-EGFP) was diluted to 150

pfu/well and incubated with peptide solution in 10% FBS/DMEM containing 0–1% DMSO for 30 min at room temperature. Vero/hSLAM cells were then infected with the inhibitor-treated MeV-EGFP for 1 h at 37 °C. The supernatant, including the virus, was replaced with 100 μM FIP (Peptide Institute, Inc. cat#4092) solution in 0–1% DMSO in 10% FBS/DMEM, and EGFP-positive cells were counted under fluorescence microscopy (BZ-X710, keyence) after incubation for 48 h at 37 °C. The entry inhibition rate was calculated as 100 − (the number of sample EGFP positive cells − the number of cell-only control EGFP positive cells) / (the number of viral control EGFP positive cells − the number of cell-only control EGFP positive cells) × 100. This experiment was performed in triplicate. IC_{50} values were calculated using parameters determined by fitting the plot to a four-parameter logistic regression model (Hill equation) implemented in Python (Matplotlib and SciPy libraries). The equation used was: $y = d + (a - d) / (1 + (x / c)^b)$. x, y, a, b, c, and d represent peptide concentration, fraction of infected cells, maximum response, Hill coefficient (degree of cooperativity), IC_{50}, and minimum response, respectively. Curve fitting was performed using the curve_fit function from the SciPy library, and IC_{90} values were calculated using the derived Hill coefficient and IC_{50} values.

Virus-mediated cell-to-cell fusion assay. Vero/hSLAM cells were cultured in 96 well plates (Thermo scientific cat#167008) overnight at 37 °C. Vero/hSLAM cells were then infected with MeV-EGFP (200 pfu / well) for 1 h. The supernatant, including the virus, was replaced with 10% FBS/DMEM with or without 1, 10, 100 μM FIP or a FIP derivative. The images of cell-to-cell fusion were captured by fluorescence microscopy (BZ-X710, keyence) after incubation for 48 h at 37 °C.

Cell-to-cell fusion assay using wild-type MeV-F plasmid. Vero/hSLAM cells in 96-well plate were transfected with 0.01 μg pCA7-ICH, 0.0375 μg pCA7-ICF and 0.3 μg pCA7 plasmid encoding EGFP (pCA7-EGFP) using VeroFect (0.695 μL, OZ Biosciences) in 10% FBS/DMEM when the confluency of the cells was about 90%. FIP or an FIP derivative in DMSO was added in each sample to 100 or 0.5 μM as the final concentration. The cells were incubated at 37 °C for 24 h and observed on a fluorescence microscope (BZ-X800, keyence).

Cell-to-cell fusion assay using MeV-F E471S mutant. In 96 well plates, Vero cells were transfected with pCA7-ICH (0.04 μg), pCA7-ICF-E471S (0.15 μg) and pCA7-EGFP (0.2 μg) encoding wild-type H, E471S mutant F protein and EGFP, using Verofect (0.78 μL, Funakoshi Co., Ltd.) in 10% FBS/DMEM when the confluency of the cells was about 90%. FIP or an FIP derivative in DMSO was added in each sample to 100, 10, or 1 μM as the final concentration. The cells were incubated at 37 °C for 24 h and observed on a fluorescence microscope (BZ-X800, keyence).

Surface plasmon resonance measurement (SPR). SPR measurements (single cycle kinetics) were performed on Biacore T100 (GE Healthcare) using the Series S Sensor Chip CM5 (Cytiva). FIP-NH-EG-K and FIP-G3r-NH-EG-K were immobilized on the sensor chip using amine coupling chemistry (7 min 0.05 M NHS/0.2 M EDC·HCl, 162 s (FIP-NH-EG-K) or 150 s (FIP-G3r-NH-EG-K) 1 mM peptide in 10 mM borate/1 M NaCl buffer (pH = 8.5), and 7 min 1 M ethanolamine). The immobilized amounts of FIP-NH-EG-K and FIP-G3r-NH-EG-K were 482 and 241 RUs, respectively. MeV-F (12.5, 25, 50, 100, and 200 nM for FIP-NH-EG-K and

1.563, 3.125, 6.25, 12.5, and 25 nM for FIP-G3r-NH-EG-K) was injected onto the peptide-immobilized sensor chip at a flow rate of 60 μL/min (25 °C, running buffer: HEPES (pH 7.4) containing 0.05% Tween 20, contact time: 3 min, final dissociation time: 10 min). The sensorgrams were fitted to a 1:1 binding model for kinetic analysis. For the curve fitting, about 180 s of the beginning of the final dissociation data was used. The rest of the dissociation data was not used for the curve fitting. The plots were fitted to a steady-state affinity model for affinity analysis.

Docking simulations. Docking simulations were performed using AutoDock Vina 1.1.2[7,8]. MeV-F was obtained from the Protein Data Bank (PDB ID: 5yzd) and pre-processed by removing water molecules and adding hydrogen atoms. Ligands were prepared and optimized using Avogadro, then converted to PDBQT format using AutoDockTools. The docking grid box was centered at coordinates (x: −26.322, y: −22.844, z: −36.233) with dimensions (30 Å x 30 Å x 30 Å). Docking was executed with an exhaustiveness parameter of 10. The top 20 binding poses were ranked by the Vina scoring function within an energy range of 3 kcal/mol. Docking results were analyzed with the Protein-Ligand Interaction Profiler[9] and visualized using UCSF Chimera[10]. The results presented in figures 4a and 4c were selected based on the criterion of the smallest RMSD for the atoms constituting Z-D-Phe-Phe of the FIP in the crystal. The following three PDB files, containing the docking poses, are provided as Supplementary Information:

1. MeV-F_predocking.pdb: The MeV-F structure obtained by removing the FIP structure from 5yzd.pdb
2. FIP_with_shifted-residues.pdb: The FIP structure along with the MeV-F residues that shifted during the docking simulation.
3. FIPG3rNH2_with_shifted-residues.pdb: The FIP-G3r-NH2 structure along with the MeV-F residues that shifted during the docking simulation.

Stability in serum. 1 μM FIP or FIP-G3r-NH_2 was prepared at 1 μM in 10% FBS/PBS in a screw tube. Tubes were sealed by Parafilm and incubated at 37 °C. After 1, 3, 7, and 24 h, 50 μL of each sample was removed to a 1.5 μL tube and quenched by 200 μL acetonitrile. After vortexing, each sample was centrifuged at 21,000 g for 30 min at 4 °C. 150 μL of the supernatant was transferred to another 1.5 mL plastic tube. 2.1 μL of DMSO and 87.9 μL of ultrapure water were added to each sample. Samples were filtered using 0.45 μm filter before quantification by LC-MS. [C11K]tigerinin-1R[11] served as a positive control to assess the proteolytic activity of the serum. It degraded below detectable limits within one hour of incubation in 10% FBS/PBS, confirming serum activity.

Serum adsorption. The experiment was conducted according to the literature[12] with some modifications to the procedures. RED (rapid equilibrium dialysis) Device base plate (Teflon® Materia) was placed in 20% ethanol for 10 min and rinsed twice with ultrapure water, then dried before use. The RED inserts (MWCO 8K) were inserted in the base plate after the base plate was dried. 300 μL of 1 μM FIP, FIP-G3r-NH_2 or propranolol in 10% FBS/DMEM and placed in one of the chambers (serum chamber), and 350 μL PBS (pH = 7.4) was added to the other chamber (buffer chamber). The plate was sealed and incubated at 37 °C with continuous shaking at 300 rpm on ThermoMixer™ C (Eppendorf). After 8 h, 50 μL of each post-dialysis sample from the plasma chamber and the buffer chamber was transferred to a 1.5 mL plastic tube. 50 μL of PBS was added to

the samples, and 50 μL 10% FBS/DMEM was added to the buffer samples. 300 μL of acetonitrile was added to each sample to stop the reaction. After vortexing, each sample was centrifuged at 16,000 g for 15 min. 200 μL of the supernatant was transferred to another 1.5 mL plastic tube. 3 μL of DMSO and 100 μL of ultrapure water were added to each sample. Samples were filtered using a 0.45 μm filter before quantification by LC-MS. The calculation formula is as follows: %Peptide not adsorbed by serum = (Peptide concentration in buffer chamber/Peptide concentration in plasma chamber) × 100%, %Peptide adsorbed by serum = 100% – %Peptide not adsorbed by serum. Propranolol was used as a control that is adsorbed by serum. The experiments were conducted in quintuplicate. At a significance level of $\alpha = 0.01$, one data point for FIP-G3r-NH_2 was identified as an outlier based on the Smirnov-Grubbs rejection test, and the data was discarded.

Supplementary Figures

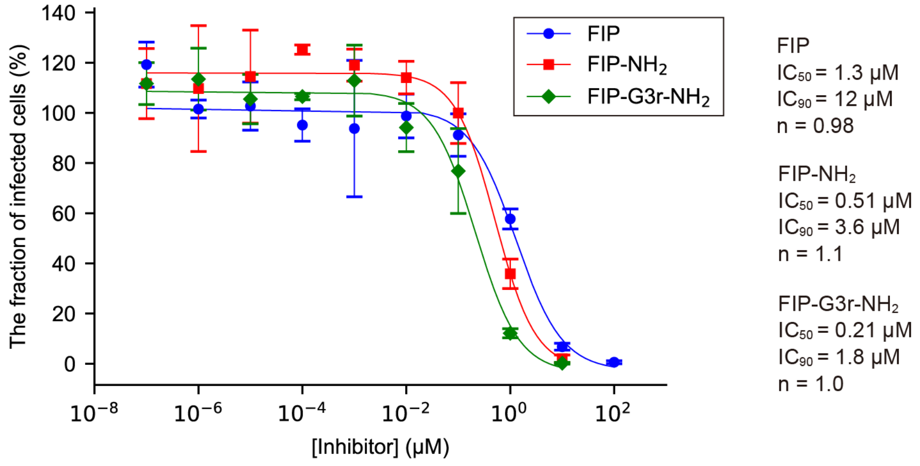

Figure S1. Inhibition curves in virus infection inhibition assay. FIP, FIP-NH_2, and FIP-G3r-NH_2 are shown in blue, red, and green, respectively. All the experiments were conducted in triplicate.

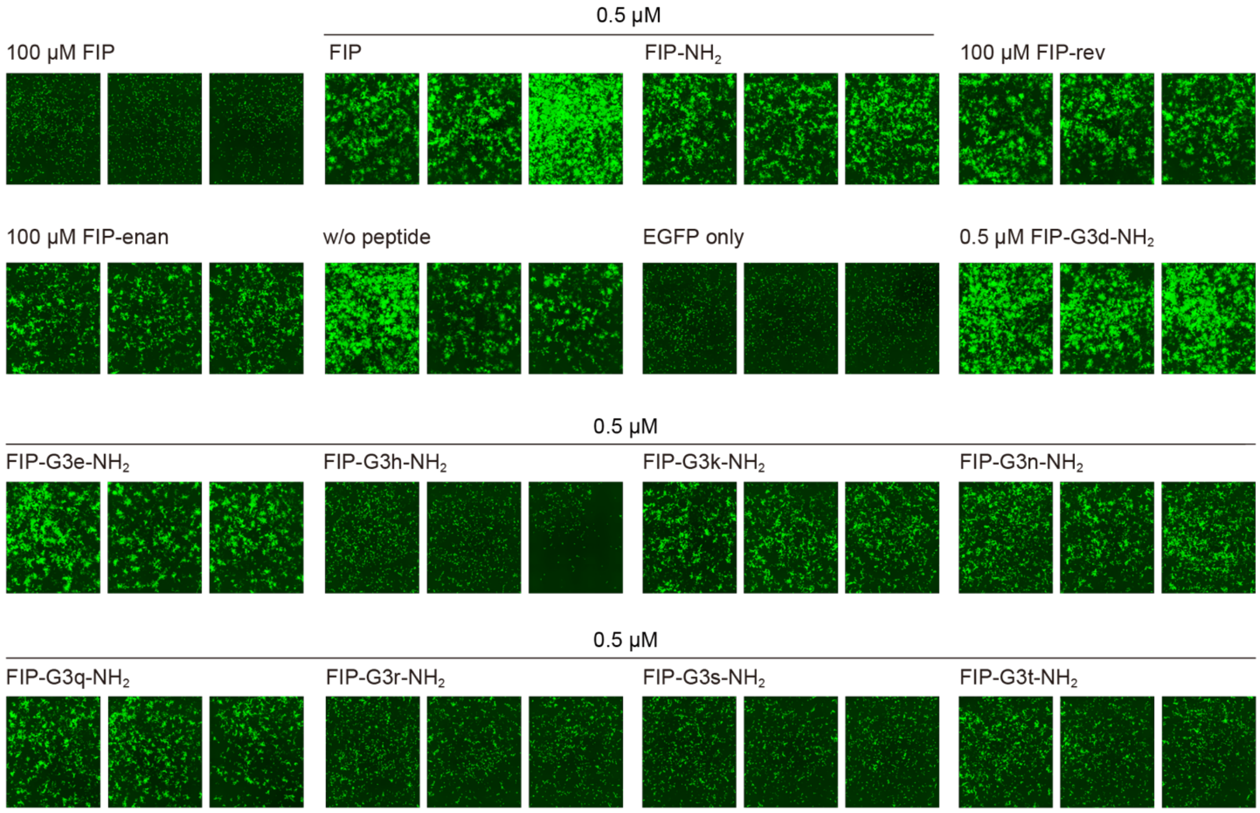

Figure S2. Cell-to-cell fusion inhibition assay of FIP and its derivatives using Vero/hSLAM cells expressing MeV-H and wild type MeV-F. The degree of cell fusion inhibitory activity was evaluated based on the area of EGFP fluorescence in Vero/hSLAM cells. All the experiments were conducted in triplicate. The "EGFP only" panel is the result of the assay with Vero/hSLAM cells transfected with only the EGFP plasmid. Other panels are the results of the assays with Vero/hSLAM cells transfected with MeV-H, MeV-F, and EGFP plasmids. The left one among the three images in each panel is used for Figure 3c and 3d.

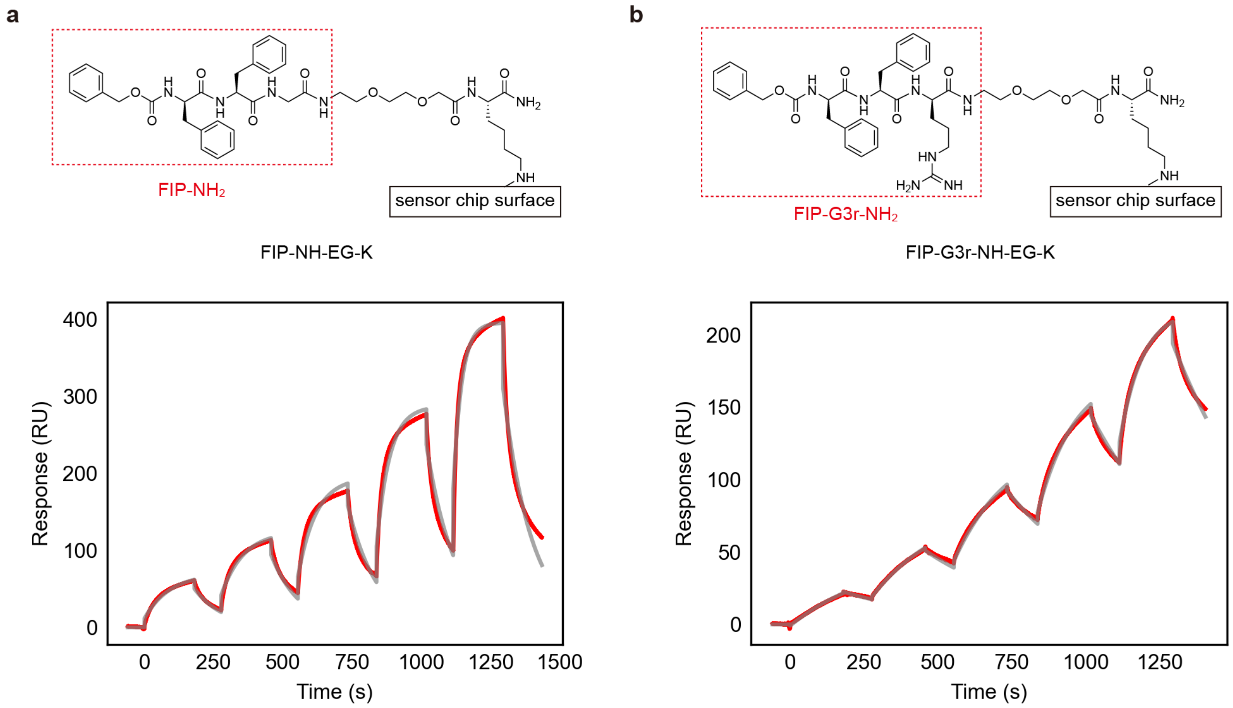

Figure S3. Binding kinetics of peptides to MeV-F measured by surface plasmon resonance (SPR) spectroscopy using Biacore T100. MeV-F was injected onto (a) FIP-NH-EG-K- or (b) FIP-G3r-NH-EG-K-immobilized CM5-chip. The sensorgrams of (a) and (b) were fitted to a 1:1 binding model. Shown in red is the raw data and in gray is the data fit. The structures of FIP-NH-EG-K and FIP-G3r-NH-EG-K are shown above the sensorgrams. The structures corresponding to FIP-NH_2 and FIP-G3r-NH_2 are highlighted by red boxes.

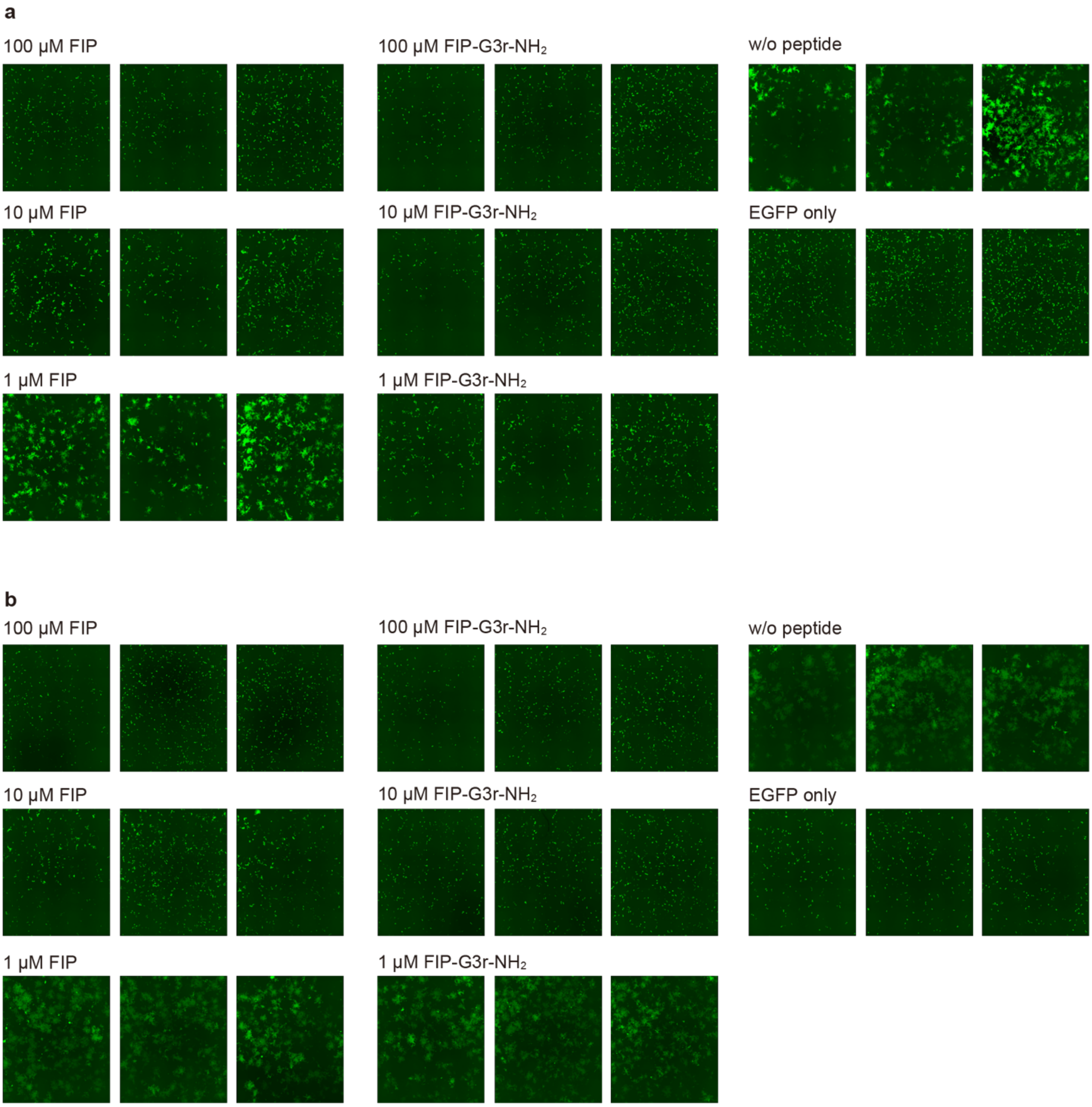

Figure S4. Comparison of the inhibitory activity of FIP and FIP-G3r-NH_2 on membrane fusion mediated by wild type MeV-F and the E471S mutant MeV-F using a cell-to-cell fusion inhibition assay. Cell-to-cell fusion inhibition assay of FIP and FIP-G3r-NH_2 using a) Vero/hSLAM cells expressing MeV-H and wild type MeV-F, and b) Vero/hSLAM cells expressing MeV-H and MeV-F E471S mutant. The degree of cell fusion inhibitory activity was evaluated based on the area of EGFP fluorescence in Vero/hSLAM cells. All the experiments were conducted in triplicate. One of the three images in each panel is used for Figure 4e.

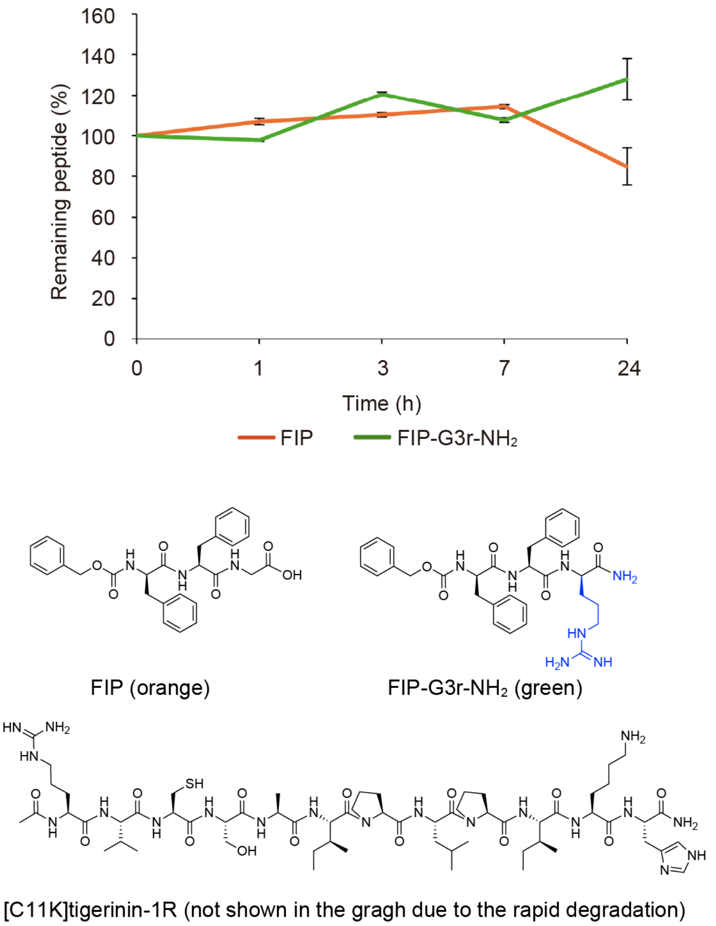

Figure S5. Stability of FIP, FIP-G3r-NH_2, and [C11K]tigerinin-1R in 10% FBS/PBS. The compounds were used at 1 μM. [C11K]tigerinin-1R is utilized as a rapidly-degrading control to confirm the proteolytic activity in serum. The control peptide was degraded rapidly and was not detected after incubation at 37°C in 10% FBS/PBS for 1 h.

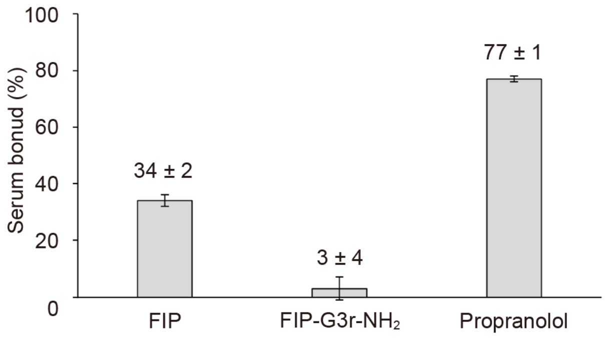

Figure S6. Serum adsorption of FIP, FIP-G3r-NH_2, and propranolol at 1 μM. Propranolol, which is known to be adsorbed to serum, was used as a control. The values and error bars of FIP and propranolol are average and standard deviations of experiments in quintuplicate. The values and error bars of FIP-G3r-NH_2 are average and standard deviations of experiments in quadruplicate.

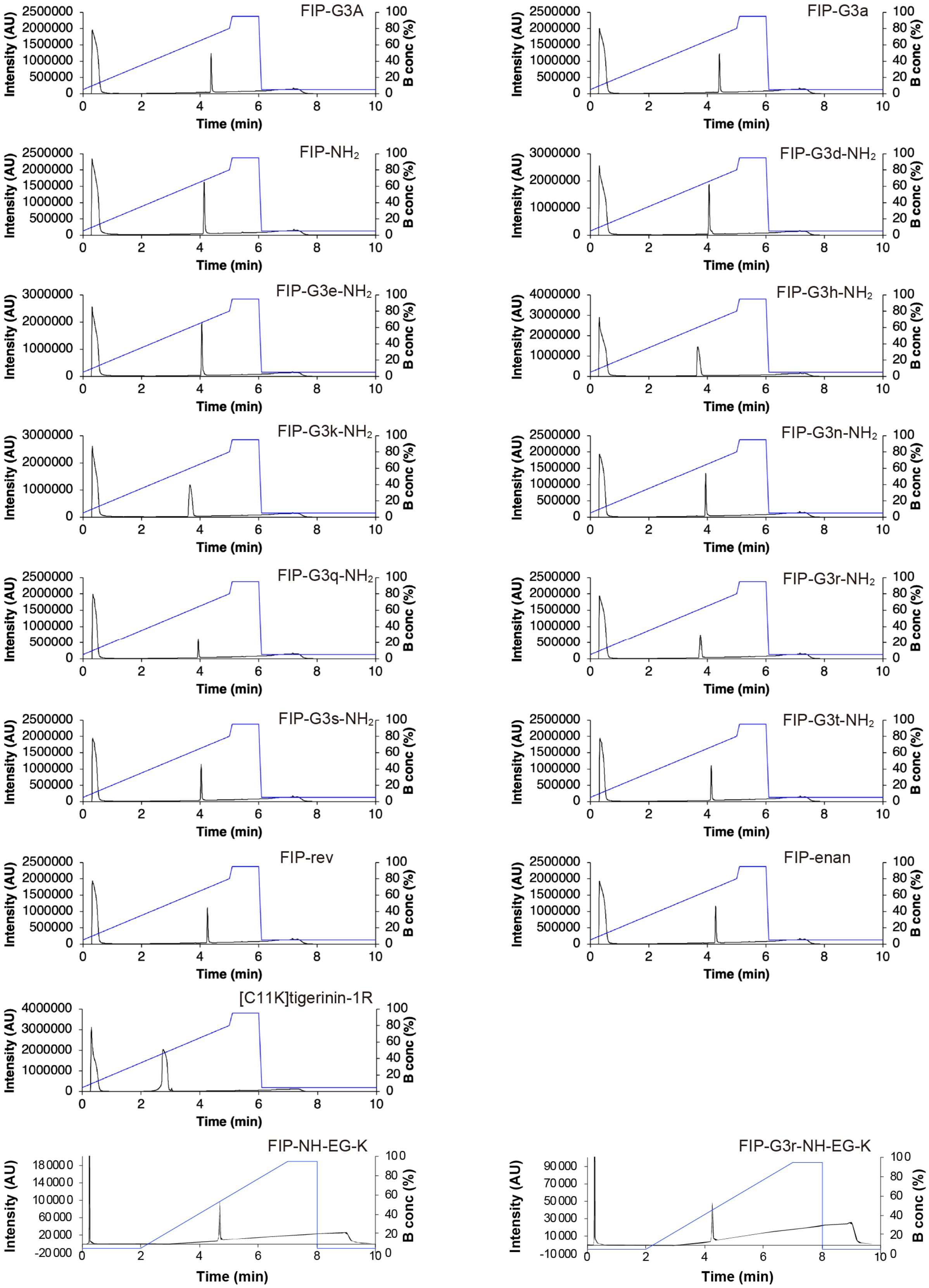

Figure S7. UV chromatograms of peptides after purification (black) and gradient curves of acetonitrile containing 0.1% TFA (blue). Compounds were monitored at 220 nm. The compounds were analyzed on a Nexera-i (Shimadzu) except for FIP-EG-K and FIP-G3r-EG-K. These two compounds were analyzed on an ACQUITY UPLC H-Class (Waters).

References

1. N. Ono, H. Tatsuo, Y. Hidaka, T. Aoki, H. Minagawa, Y. Yanagi, *J. Virol.*, 2001, **75**, 4399–4401.
2. M. Takeda, K. Takeuchi, N. Miyajima, F. Kobune, Y. Ami, N. Nagata, Y. Suzaki, Y. Nagai, M. Tashiro, *J. Virol.*, 2000, **74**, 6643–6647.
3. M. Takeda, S. Ohno, F. Seki, Y. Nakatsu, M. Tahara, Y. Yanagi, *J. Virol.*, 2005, **79**, 14346–14354.
4. N. Komune, T. Ichinohe, M. Ito, Y. Yanagi, *J. Virol.*, 2011, **85**, 13019–13026.
5. M. Kubota, K. Takeuchi, S. Watanabe, S. Ohno, R. Matsuoka, D. Kohda, S. I. Nakakita, H. Hiramatsu, Y. Suzuki, T. Nakayama, T. Terada, K. Shimizu, N. Shimizu, M. Shiroishi, Y. Yanagi, T. Hashiguchi, *Proc. Natl. Acad. Sci. U.S.A.*, 2016, **113**, 11579–11584.
6. S. Watanabe, Y. Shirogane, S. O. Suzuki, S. Ikegame, R. Koga, Y. Yanagi, *J. Virol.*, 2013, **87**, 2648–2659.
7. O. Trott, A. J. Olson, *J. Comput. Chem.*, 2009, **31**, 455–461.
8. J. Eberhardt, D. Santos-Martins, A. F. Tillack, S. Forli, *J. Chem. Inf. Model.*, 2021, **61**, 3891–3898.
9. M. F. Adasme, K. L. Linnemann, S. N. Bolz, F. Kaiser, S. Salentin, V. J. Haupt, M. Schroeder, *Nucleic Acids Res.*, 2021, **49**, W530–W534.
10. E. F. Pettersen, T. D. Goddard, C. C. Huang, G. S. Couch, D. M. Greenblatt, E. C. Meng, T. E. Ferrin, *J. Comput. Chem.*, 2004, **13**, 1605–1612.
11. X. Chen, C. Hu, Y. Huang, Y. Chen, *Int. J. Mol. Sci.*, 2018, **19**, 1–13.
12. N. J. Waters, R. Jones, W. Gareth, Bi. Sohal, *J. Pharm. Sci.*, 2008, **97**, 4586–4595.
